# Evaluating poor working conditions and health problems faced by carpet weavers in Kashmir: a qualitative analysis

**DOI:** 10.1080/17482631.2024.2421575

**Published:** 2024-11-07

**Authors:** Tanveer Ahmad Khan, Shaista Qayum

**Affiliations:** a Institute of Kashmir Studies, University of Kashmir, Srinagar, India; b Department of Higher Education, (J&K), AAAM Govt. Degree College Bemina, Kashmir, India

**Keywords:** Carpet weavers, poverty, asthma, musculoskeletal problems, stress, fatigue

## Abstract

This study provides a comprehensive exploration of the working conditions and associated health challenges faced by carpet weavers in Kashmir. The detailed examination of various factors contributes significantly to understanding the intricacies of their daily lives and provides valuable insights into an often-overlooked aspect of occupational health. The data was collected through 22 individual interviews and 5 Focus Group Discussions (FGDs). A semi-structured interview schedule was used to collect data allowing researchers to engage with participants. The results of the study emphasize that carpet weavers are not satisfied with the existing working conditions of the workshops they work in. Most carpet weavers suffer from multiple and sometimes chronic health ailments like musculoskeletal problems, respiratory problems, skin problem, fatigue and other health problems such as abdominal pain, nose problem, and anaemia owing to the unsafe working environment, long working hours, inappropriate sitting postures, poorly designed tools, exploitative circumstances, and low wages. The study recommends need for recognition of carpet weavers as vulnerable workers. The call for improved working conditions, better infrastructure, and awareness initiatives aligns with the goal of fostering a healthier and supportive environment for these workers. There is a significant need to address the identified health problems to safeguard both the carpet industry and the livelihoods of the weavers.

## Introduction

Carpet weaving is a distinctive traditional occupation in many countries like Iran, China, Turkey, India, Pakistan, Russia, Egypt, Nepal, and Afghanistan (Karimi et al., 2016). In Kashmir, this tradition flourished for a long time after Sultan Zain-ul-Abidin’s rule but showed significant revival through the efforts of Akhund Rahnuma in early 1600 (Irfan, 2011; M. I. Khan, 1978; Shah, 1992). Therefore, the origin of carpet weaving in Kashmir can be traced back to the 15^th^ century during the reign of Sultan Zain-ul-Abidin, also known as Budshah (Bhat & Singh, 2017; Shah, 1992) and weaving is carried out in homes as well as factories (Khan, 1993). The Sultan, known for his patronage of arts and crafts, is credited with introducing the craft to Kashmir (Dewan, 2008; Khan, 1978). It is believed that he invited Persian artisans to the region, who brought with them the intricate techniques and designs of Persian carpet weaving (Ahmad, 2007; Mattoo , 1988). This marked the beginning of the carpet industry in Kashmir, which soon became an integral part of the region’s cultural and economic life (Raina, 2012). By continuing the practice of carpet weaving, artisans in Kashmir help preserve this cultural heritage, passing down skills and knowledge from generation to generation (Ahmad, 1985; Ahmad, 2007; Rao, 2019).The carpet industry in Kashmir flourished significantly in the Mughal period, particularly during the reign of Emperor Akbar in the 16^th^ century (Shah, 1992). Akbar, who had a keen interest in Persian art and culture, further encouraged the craft by bringing weavers that are more skilled from Persia and Central Asia (Khan, 1978). The Mughal influence is evident in the designs and motifs used in Kashmiri carpets, with many patterns reflecting Persian aesthetics blended with Indian elements. This period is often regarded as the golden age of carpet weaving in Kashmir, where the craft reached new heights in terms of quality and artistic expression (Mehta, 2014). The 19^th^ century brought significant changes to the carpet industry in Kashmir. Under British colonial rule, the industry faced both opportunities and challenges. On the one hand, Kashmiri carpets gained exposure to international markets, particularly in Europe and the industry suffered from exploitation by British traders, who often undervalued the craftsmanship and paid low wages to weavers (Ahad, 1987; Shah, 1992). Additionally, the introduction of machine-made carpets in Europe posed a significant threat to the traditional hand-knotted carpets of Kashmir, leading to a decline in the industry during the late 19^th^ and early 20^th^ centuries (Sharma, 2005). After India’s independence in 1947, there was a renewed focus on reviving the traditional crafts of Kashmir, including carpet weaving. The government, recognizing the economic and cultural importance of the industry, initiated several measures to support the artisans (Ahmad, 1985). These included the establishment of cooperative societies, provision of credit facilities, and promotion of Kashmiri carpets in international markets (Majeed, 2018). Despite these efforts, the industry faced on-going challenges, such as political instability in the region and competition from cheaper alternatives (Mohsin & Muzaffar, 2024). However, the post-independence period also saw resurgence in demand for authentic, high-quality Kashmiri carpets, both domestically and internationally (Kaul, 2017). In recent years, the carpet industry has continued to evolve, adapting to modern market demands such as competitiveness, capacities, delivery time, and standardization while striving to preserve its traditional craftsmanship (Srivastava & Goswami, 2007). The introduction of contemporary designs, alongside the traditional motifs, has helped the industry appeal to a broader audience. Moreover, efforts were made to obtain Geographical Indication (GI) tags for Kashmiri carpets, which help protect the authenticity and uniqueness of the product in global markets (Bhat & Singh, 2019). A geographical indicator (GI) is a label applied to products that are identified by their specific geographical origin and possess characteristics or a reputation unique to that location (Karobi, 2022). Despite challenges, the industry remains a vital part of Kashmir’s cultural heritage and economy (Mir, 2020).

The carpet industry employs nearly 2.2 million full and part-time weavers, with 8.5 million people whose livelihood is dependent on hand-woven carpets (Sobhe, 1997). Likewise, in Kashmir, the carpet industry employs nearly 100,000 weavers, including both genders, majority of them belonging to rural areas (Irfan, 2011). The labour-intensive nature of carpet weaving means that it employs a large number of artisans, including a significant proportion of women (Majeed & Swalehin, 2021). The sector also creates indirect employment through related industries, such as raw material suppliers, dye makers, and traders (Akhtar et al., 2017; Majeed & Swalehin, 2021). According to a study by Ahmad (2018), the carpet industry in Kashmir employs over 200,000 people, making it a critical source of income for the region. Therefore, carpet weavers constitute a significant percentage of the population in Kashmir, and their contribution to economic development cannot be overemphasized (Nengroo & Bhat, 2017). Thus, carpet weaving is a major contributor to Kashmir’s economy, especially through exports. The export of these carpets brings substantial revenue, supporting not only the weavers but also the broader economy of Kashmir (Bano, 2016; Raina, 2009). In 2019–2020, the total export value of carpets from India was around $1.37 billion, with a significant portion coming from Kashmir (Export Promotion Council for Handicrafts, 2020). The main export markets for Kashmiri carpets are USA, UK, Germany, Canada and Australia (Majeed & Swalehin, 2020).

Weaving skills like the cultivation of the silk or wool, setting the loom, reading the *taleem*,^1^ deciding weaving patterns, designing, tying, and dyeing of threads, using acid, chrome, and de-looming and re-looming are transmitted from *ustaad*^2^ to *shagird*^3^ (master-apprentice system) (Saraf, 1987). *Taleem* is read and interpreted by weavers *(kaalbaaf)*^4^ individually and communicated in a cryptic trade-language among their teams to weave the design (Kaur, 2023). More importantly, among these skills, designing, coding, and weaving are not only physical activities but are also cognitive ones, involving a wide range of cognitive accomplishments achieved by weavers from experience (Kaur, 2018). Khan (1993) argues that cognitive activity in the everyday world occurs in a socio-cultural setting and therefore, is defined, interpreted and supported socially. In practice, the *taleem* fulfils several cognitive roles, including serving as an information repository, providing computational support, encoding temporal actions, functioning as a communication device, distributing cognitive labour, and carrying cultural heritage (Kaur, 2018). Manual and digital are the two main design settings used during the design phase. In the manual approach, the carpet designer *(naqash)* creates the design on graph paper and uses colour codes to add specific colours to the pencil sketches (Ahmad, 1985). The coded graph is then passed to the *talim*-writer (*talim*-guru or *talim nawis*), who systematically writes practice-specific symbols on long strips or rolls of paper, including the colour codes and the number of cells or knots (Cross, 1991; Kaur, 2017, 2018; Tramboo, 1998). This division of cognitive labour involves several participants-designers, coders, and weavers-each working with distinct artefacts in different social contexts, ranging from households to professional karkhanas (factories) (Kaur, 2018). The primary artefact in this process is the *talim*, which transitions between task domains and performs various cognitive functions for different actors. These functions include representing and communicating information for all participants, encoding temporal weaving actions for coders and weavers, coordinating team activities, establishing roles and hierarchies for weavers, and preserving cultural heritage for the community (Indo-Global Social Service Society, 2015–2016; Kaur, 2018).The process is quite laborious from beginning to end, as most of the weavers prefer hand weaving (Community, 2016), which leads to many health problems among carpet weavers (Noorbala, 2008; Parida, 2019). Raju and Rao (2014) found that 90% of weavers experience health problems such as asthma, tuberculosis, poor vision, and hernia. Tramboo (1998) reported that children working in the carpet industry commonly suffer from respiratory issues, body aches, joint pains, finger pain, weak eyesight, general weakness, and a lack of appetite. According to Mattoo et al. (1986), the primary complaints among carpet weavers include headaches, blurred vision, back pain, abdominal pain, various injuries, joint pain, dysentery, unexplained fevers, limb pain, respiratory infections, dermatitis, and chilblains. This worsening condition is not only a problem in Kashmir but is also a global issue (Khan et al., 2022).

Despite the significant role of carpet weavers in the economy and environmental protection, they are exposed to unsafe conditions in this job and receive low wages. The carpet-weaving community is mainly concentrated in unclean spaces and lacks proper sanitation facilities. Even though standard cleanliness is maintained inside the house, it does not conform to basic standards to avoid diseases and promote good health (Indo-Global Social Service Society, 2015–2016). Working conditions of weavers and occupational diseases have a closer association with each other. Previously conducted studies confirmed that various occupational diseases such as eyesight deterioration, fatigue, respiratory diseases, gastrointestinal problems, bodily injuries of the upper extremity and other health issues are associated with the carpet weaving industry (Gada et al., 2024; Joshi & Dahal, 2008; Parida 2019; Shrestha et al., 2008; Tramboo, 1998; Thomas, 2011). These diseases are mostly induced by lack of proper maintenance, overcrowded production units, airborne contaminants, inhaling of weaving material, inappropriate work posture, inevitable use of hookah, physical exertion, stressful work, harmful components inherent in materials used by the weavers, and working in poorly ventilated and illuminated conditions (Gani & Shah, 1998; Joshi et al., 2011; Khan et al., 2022; Parida, 2019; Raju & Rao, 2014). Weavers across the globe are victimized not so much by traditional labour practices as by the capricious cycles of capitalism (O’Neill, 2004), which made professional *taleem* writers hard to find nowadays because of computer aided designs (CAD) on the scene (Kaur, 2018). Taleem-copyist (nakal-nawis), used to exist for copying *talims*, but it vanished with the advent of photostat and CAD in 1990s (see Kaur, 2023). Moreover, carpet weavers are trapped in a vicious cycle of poverty and are vulnerable to different health issues and poor socio-economic conditions. Although some empirical studies have been conducted on health issues faced by carpet weavers in Kashmir, not a single study has been conducted on finding an association between working conditions and health problems of carpet weavers. In response to this evidence gap, this study aims to contribute to the literature on the health status of carpet weavers in Kashmir by providing insights on the overlooked working condition and health problems faced by them in different workstations. The study began with the questions on general information like age, gender, work experience, working conditions, wages, and challenges associated with weaving tools, followed by some probing questions to understand participants weaving experiences.

## Method

### Approach

Abductive research helps researchers develop a theoretical understanding of the contexts and perspectives of the people they study, focusing on how they perceive the world (see Conaty, 2021). The key step in abduction is that, after describing and understanding the world from the participants’ perspectives, researchers must create a social scientific account based on those viewpoints. Unlike pure induction, abduction emphasizes using participants’ worldviews to form the explanation (Bryman, 2012). For example, when studying carpet weavers’ health problems, researchers might ask why they accept work that seems to offer little benefit, aiming to deeply understand their perspectives. We used abductive research approach to understand participants’ social world: their construction of reality, their way of conceptualizing and giving meaning to their social world, and their tacit knowledge that can be discovered from the accounts provided by them (Blaikie, 2000, p. 25). For the present study, this means pinning the voices of carpet weavers at the core of the investigation to understand their working conditions, their meaning of work, the future of their occupation, the health problems they experience, and how they view the role of policies in promoting health equality.

### Sampling and participant recruitment procedure

According to Krueger and Casey (2000) recruitment through FGDs can be expensive, challenging, and a topic of vigorous debate. Therefore, to overcome the issue, we used FGDs and face-to-face interviews correspondingly. Carpet weavers aged between 15 and 51+ were eligible to participate in the study. Other significant criteria included presently practicing weaving; having been diagnosed with an illness because of carpet weaving; must be a dependent weaver; and being ready to participate. Potential participants were recruited through three channels; with the help of two key informants who belonged to the non-weaving community, by visiting different weaving workshops, and snowball sampling. Three blocks Sherpathari, Wakura, and Kangan of district Ganderbal were selected for the study through purposive sampling. These blocks have been studied because, unlike other parts of the Ganderbal district, carpet weavers are predominantly concentrated in these regions. Additionally, these blocks were selected due to the high concentration of government training centres. In total, we conducted 5 FGDs and 22 individual interviews. The participants in FGDs did not include those who participated in the individual interviews. All interested participants were informed about the purpose of the study. The researchers made sure that the participants took part voluntarily. In case of any discomfort/unease, they were free to decline, withdraw, or not respond to any questions. Participants also gave explicit oral consent for their pictures to be used in the study. After the first meeting, the date and venue for interviews were decided mutually. Their rights were protected through anonymity and confidentiality. Both aspects were managed by safeguarding the participants’ identities. Pseudonyms were used throughout the study to maintain confidentiality. The information collected, including photos, audios, videos, and field notes were stored securely. The data was not shared with anyone except the participants who wished to view their photos and videos. Two videos, capturing intense arguments among four weavers in a loom shed, were deleted at the participants’ request. Our study did not require any formal or institutional approval.

### Data collection

Qualitative techniques were given priority to gather information from the field. The employed methods helped us to record the carpet weavers’ voices and understand their experiences from their point of view. Numerous approaches, such as observation, ethnographic writing, semi-structured interviews, oral histories, and group discussions, are used in qualitative research (Brockington & Sullivan, 2003; see Glaser and Strauss, 1967). These techniques produce abundant, explicative, and frequently multifaceted data (Kielmann et al., 2012). We collected most of the qualitative data through 22 individual, face-to-face interviews with weavers followed by 5 FGDs between July and November 2019. Each Focus Group had a minimum of four and a maximum of seven participants. During FGDs and interviews, both direct and indirect observations were employed. Direct observation, for instance, was employed to observe what was happening at the workplace. Interactions, attitudes, and gestures were used to accomplish this. For indirect observation, clues, traces, and artefacts such as hanging of amulets, weaving balls, saint’s images, shrines, and tablets left in the strip were employed. In most of the cases, notes were taken down while the weaving process was observed. Particular attention was paid to how quickly the carpet weavers tied the knots. Each FGD was conducted with the prior consent of the owners and workers (carpet weavers). FGDs and interviews were performed during working days and occasionally on holidays. Each FGD and interview was approximately completed in two to three hours. FGDs and interviews began with a simple inquiry: please tell us something about the nature of the weaving occupation, wages, working hours; how is weaving different from other occupations; does weaving lead to health problems, followed by probing questions to elicit further information on the topic. Pauses were used throughout the interviews to allow participants to take their time and respond comfortably. The questions were written in English for research purposes but were asked in Kashmiri, the participants’ mother tongue, to get complete and authentic information from them. The qualitative material obtained through narratives, field notes, and recordings was documented to ensure that everything was noticed after the fieldwork. Non-observation (done by the first author) also helped the researchers with respect to gathering additional qualitative data. Field notes also served as a supportive data source for this study (see Figure 3a,d). Field notes were typed after each interview and later analysed and compared with the transcripts of the interviews and FGDs. We continued gathering data until we reached a point where no new insights were emerging from the participants. After conducting the 20^th^ interview, we observed that no additional information was being revealed. Consequently, we decided to conclude our data collection after interviewing 2 more participants, making a total of 22 carpet weavers who were interviewed. The names of participants in individual interviews are represented as Participant 1, Participant 2 and so on. FG1, FG2, FG3, FG4, and FG 5 denotes focused group interviews of males and FG1*, FG2*, FG3*, FG4* and FG5* denotes focused group interviews of females.

### Data analysis

Each FGD and interview was carefully translated from Kashmiri to English to capture the core of the question. Each recorded interview was carefully listened to up to 3–5 times before being translated to ensure no crucial material was missed. Two overarching themes emerged from the data analysis. These included carpet weavers’ health problems and their satisfaction with their working conditions. To extract the major themes from the data, Braun and Clarke (2006) proposed a thematic analysis, which was meticulously followed. It was described as a technique for finding, examining, and reporting patterns (themes) in data. Using thematic analysis as a constructionist approach, we looked at participants’ working experiences, working environments, and associated health issues. According to scholars like Kiger and Varpio (2020) and Braun and Clarke (2016), thematic analysis is appropriate for interpreting experiences, thoughts, or behaviour’s within a data set. We used the fundamental procedures Braun and Clarke (2006) recommended to identify the main themes. We first became familiar with primary data, which was transcribed by repeatedly and actively reading the data and we took note of the initial concepts (see Nowell et al., 2017; Thorne, 2000). The data set included interviews, focus groups, recorded observations, field notes, images, and videos. By transcribing the audio data, we became familiar with it. Second, we identified the initial list of concepts in the data by marking them, and then we searched for what was intriguing about those ideas. We painstakingly framed the first manifest and latent codes using such notions. We made notes on possible data items of interest and queries and looked for links between data items and other initial ideas (see Braun & Clarke, 2006; Kiger & Varpio, 2020. The entire coding was done manually. This step was followed by searching for themes in which we gathered all relevant data for each potential theme. We developed themes through code analysis, contrast, and combination. Because we explicitly retrieved the themes from the coded data, they were more closely tied to the original data and reflective of the entire data set (Braun & Clarke, 2006). The study employed inductive analysis in this way. Following this, we reviewed the themes to which we had assigned data for each theme, deleted the themes for which there was insufficient data, and added, combined, and divided some remaining themes. In order to properly reflect and capture coded data, data extracts were rearranged at this step and themes were adjusted (see Braun & Clarke, 2006). When everyone among us was content with the updated themes and agreed that they appropriately covered all of the coded data, we called off this process. We kept thorough notes and memoranda throughout the process on our study design, decisions regarding how we framed themes, amended and eliminated them, and other pertinent information. Memos enable researchers to connect disparate topics and produce an audit trail that strengthens the reliability of their findings, as illustrated by Nowell et al. (2017). Another crucial stage in our investigation was defining and naming themes. At this point, we improved the details of each theme and the overall narrative the analysis conveys, giving each theme a different name and meaning (Bauman & Clarke, 2006). Each theme was given a definition and a narrative explanation, along with a discussion of its relevance to the overarching research question (Braun & Clarke, 2006). The final report’s theme titles were examined to ensure they were succinct and illustrative (Braun & Clarke, 2006). Producing the report was the final step in our analysis. At this stage, we did a final analysis of selected extracts, relating the analysis to the research question and literature and produced a scholarly discussion. This final step involved writing the final analysis and description of the findings (Braun & Clarke, 2006). As opposed to being a “separate stage”, King (2004) defined the last step of presenting findings as a “continuation” of the analysis and interpretation that has already taken place (p. 267). We directly quoted participants to summarize the data and provide in-depth justifications and responses to submitted study questions. The direct data extracts contain adequate context to understand participants’ everyday life in the weaving occupation.

### Results

A total of *n* = 22 carpet weavers, aged 15 to 51+ years old, participated in the study. Most participants lived in rural areas of district Ganderbal (*n* = 20, 90%) (Table I). Among participants, the number of male (*n* = 13, 59%) was slightly higher than female (*n* = 9, 40.9%). Most participants (*n* = 15, 68.18%) were married, *n* = 6 (27.27%) were unmarried, and *n* = 1 (4.55%) was widowed (Table I). Regarding education, most participants (*n* = 15, 68.18%) were illiterate, and only a few (*n* = 4, 18.18%) studied up to the primary level, followed by even fewer (*n* = 3, 13.64%) who have completed a secondary level of education (Table I). Literacy was found high among male carpet weavers than female carpet weavers. The lowest number of years spent in carpet weaving was identified as 5–9 years (*n* = 3, 13.64%), and the highest number of years spent by the participants was identified as 10–19 years (*n* = 11, 50.00%). 30+ years were only spent by some (*n* = 2, 9.09%) participants (Table I). There is also a close association between occupational illness and the number of years spent in weaving. It was observed that participants who had spent more years in carpet weaving reported more illnesses than those who had spent fewer years in weaving. Participants also reported long working hours as a significant cause of their illness. Most (*n* = 12, 54.55%) reported working 9–12 hours daily, and more than 17 hours of daily work was reported by one of the respondents (*n* = 1, 4.54%). Most of the participants (*n* = 14, 63.64%) had monthly income > 5000, and only one (*n* = 1, 4.54%) earned < 15000 rupees monthly (Table I). In terms of place of work, most (*n* = 13, 59.10%) worked at the workshop and the rest (*n* = 9, 40.90%) worked in their respective homes (Table I). Participants experienced a range of health problems such as musculoskeletal problems (*n* = 22, all the male respondents (13) constituting 59.09% and 9 female respondents constituting 40.90), respiratory problems (*n* = 16, 10 male respondents constituting 62.50% and 6 female respondents constituting 37.50%), skin problem (*n* = 15, 9 male respondents constituting 60%and 6 female respondents constituting 40.%, eye problem (*n* = 12, 7 male respondents constituting 58.33% and 5 female respondents constituting 41.66%), ear problem (*n* = 10, 6 male respondents constituting 60% and 4 female respondents constituting 40%), fatigue (*n* = 16, 9 male respondents constituting 56.25% and 7 female respondents constituting 43.75%, stress (*n* = 17, 12 male respondents constituting 70.58% and 5 female respondents constituting 29.41%), injuries (*n* = 8, 5 male respondents constituting 62.5% and 3 female respondents constituting 37.5%), and other health problems such as abdominal pain, nose problem, anaemia etc.) (*n* = 6, 4 male respondents constituting 66.66% and 2 female respondents constituting 33.33%) (Table III). Participants linked these problems with the poor working conditions, the changing nature of weaving tools, and the exploitative nature of the behaviour of the *wastas.*

**Table I.** Socio-demographic and occupational characteristics of the participants (*n* = 22).

Variable		Male (n = 13)		Female (n = 9)		Total (N = 22)	
		F	(%)	F	(%)	F	(%)
Age	15–30	3	23.08	2	22.22	5	22.73
	31–50	6	46.15	5	55.56	11	50.00
	51+	4	30.77	2	22.22	6	27.27
Marital status	Married	9	69.23	6	66.67	15	68.18
	Unmarried	4	30.77	2	22.22	6	27.27
	Widowed	0	0.00	1	11.11	1	4.55
Education	Illiterate	8	61.54	7	77.78	15	68.18
	Primary	3	23.08	1	11.11	4	18.18
	Secondary	2	15.38	1	11.11	3	13.64
Years in carpet weaving	5–9	1	7.69	2	22.22	3	13.64
	10–19	6	46.15	5	55.56	11	50.00
	20–29	4	30.77	2	22.22	6	27.27
	30+	2	15.38	0	0.00	2	9.09
Daily working hours	5–8	1	7.69	3	33.33	4	18.18
	9–12	7	53.85	5	55.56	12	54.55
	13–16	4	30.77	1	11.11	5	22.73
	17+	1	7.69	0	0.00	1	4.54
Monthly income (in Rupees)	5000	6	46.15	8	88.89	14	63.64
	5001 -10,000	4	30.77	1	11.11	5	22.73
	10001 -15,000	2	15.38	0	0.00	2	9.09
	<15001	1	7.69	0	0.00	1	4.54
Size of household	1–4	1	7.69	0	0.00	1	4.54
	5–8	5	38.46	3	33.33	8	36.36
	8+	7	53.85	6	66.67	13	59.10
Place of work	Home	2	15.38	7	77.78	9	40.90
	Workshop	11	84.62	2	22.22	13	59.10

source: fieldwork

N (number), F (frequency), % (percentage)

#### Satisfaction with the working conditions

Satisfaction with the working conditions at the workshop determines the health conditions of the workers. Most of the participants interviewed were not satisfied with the working environment of their workshops. The satisfaction with the working conditions was analysed through different parameters such as seating arrangement, lighting arrangement, cleanliness, designing of tools, *taleem*, and relationship with *wastas*. The satisfaction with the working conditions was analysed through parameters shown in Table II:

**Table II.** Level of satisfaction with the working conditions among carpet weavers.

Working condition	Carpet weavers (n=22)									
	Very satisfied		Somewhat satisfied		Neutral		Somewhat dissatisfied		Very dissatisfied	
	F	%	F	%	F	%	F	%	F	%
Seating arrangement	1	4.54	3	13.63	2	9.09	5	22.73	11	50.00
Lighting	2	9.09	2	9.09	5	22.73	4	18.18	9	40.91
Cleanliness	2	9.09	5	22.73	3	13.63	5	22.73	7	31.82
Design of tools	4	18.18	4	18.18	0	0.00	6	27.27	8	36.36
Computerized taleem	0	0.00	2	9.09	4	18.18	3	13.63	13	59.10
Relation with wasta	3	13.63	1	4.54	5	22.73	6	27.27	7	31.82

Source: *Fieldwork*.

N (number), F (frequency), % (percentage)

### Experiences with seating arrangement

**Figure 1.**
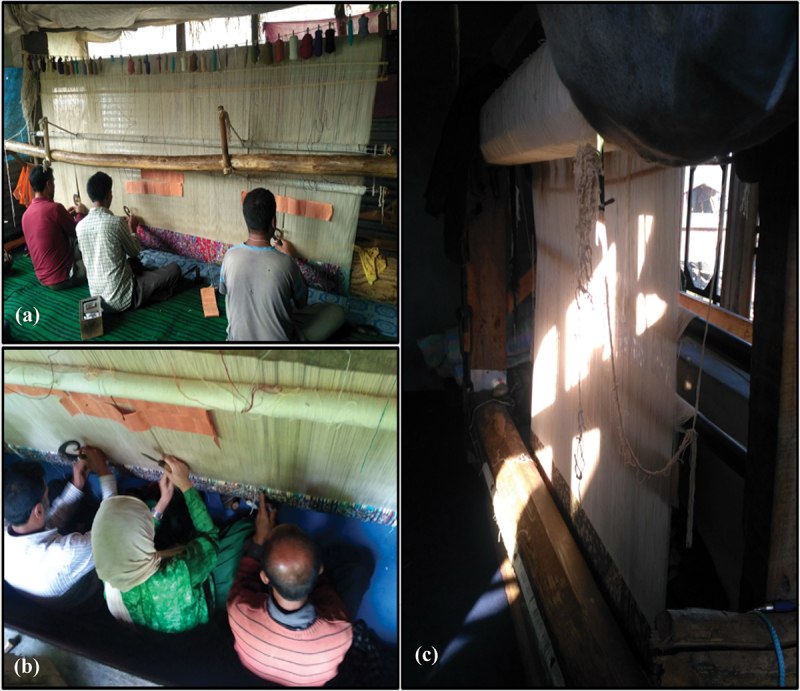
(a) represent carpet weavers work with folded legs; Figure (b) inadequate space between male and female weavers; Figure (c) shows even the mid-day looks like an evening.

**Source**: Field Work

Most of the participants were extremely dissatisfied with the seating arrangement of their workshops (Table II). During participant observation and interviews, we noted that, in most cases, participants were supposed to sit on the floor due to lack of space in the workshops. Only a few had elevated benches, which were not so helpful to the participants. In such types of seating arrangements, carpet weavers could not keep their legs straight and were forced to sit with folded legs during long working hours (see Figure 1a). The low seating arrangement with no back support also made it difficult for them to weave properly, and because of inappropriate sitting postures, they suffered from many musculoskeletal problems.

We sit on the floor to weave the carpet. There is no provision for a backrest as we are supposed to sit in the middle and thus away from the walls of the room. If I wish to keep my legs straight for some time, there is no space for it. I have to sit with folded legs the whole day. **FG1**

Though rarely cited as the problem, during interviews, the female participants expressed concern with respect to the continuous touching of the body parts while sitting with the male workers (see Figure 1b). We also noted during participant observation that there was no or very little space between carpet weavers because of the over crowdedness in the workshops.

Touching with the male carpet weaver has become normal for me. I work with three other male carpet weavers, and I occupy the third seat. Therefore, I have to sit between two male weavers for almost 7 hours a day. Initially, it was very uncomfortable for me, but I was not able to talk to anyone about it. I still feel awkward and do not like it, but I have to bear all this for my children. **Female Participant 3**

The inadequate seating arrangement significantly hinders the comfort and effectiveness of the participants during their workshops. The lack of proper seating not only affected their ability to work efficiently but also contributed to physical discomfort and potential long-term health issues. Addressing these seating concerns should be a priority to improve both the immediate experience and overall well-being of the participants.

### Experiences regarding lighting arrangement

The lighting arrangement of the workshops was another aspect with which most of the participants showed extreme dissatisfaction. Most of the rooms or sheds where the weavers worked had only one small window, making them look darker even during the day. As a result, most participants revealed that they are forced to work in dark rooms under low light (see Figure 1c). Poor lighting facilities and frequent power cuts added to the already existing lighting problem and, over time, caused eye fatigue, headaches, and poor posture.

There is a small window in the room where we work. For most of the daytime, the room remains dark. In addition, only one electric bulb provides light to the whole room. In such low light, we have to focus more while weaving, which creates problems in our eyes. **Male Participant 1**

When power cuts occur, the day seems to turn into night, and due to darkness, if by chance the weaving instrument gets misplaced, it is hard to find it. To save our lives from illness, we request owners and government officials to install solar panels for our workstations. FG2*

These narratives indicate that participants working in such conditions expressed significant dissatisfaction with the lighting arrangements in the workshops, which greatly affected their working conditions. In addition to inadequate natural light, the poor lighting infrastructure and frequent power outages exacerbated the problem. Over time, these conditions led to adverse effects on the participants’ health, including eyestrain, headaches, and poor posture, further contributing to their discomfort and reducing overall productivity.

### Experiences related to cleanliness of the workstations

The majority of the participants were extremely dissatisfied, followed by somewhat dissatisfied with the cleanliness of the rooms. The participants revealed that the rooms are cleaned twice a day (in the morning and the evening) with no provision of cleaning in between. Continuous weaving generated dust and fibre particles that remained in the air for the whole day. With inadequate ventilation and cleaning, the contaminants are trapped in the rooms and create many health problems like eye irritation, allergies, and difficulty in breathing for the weavers.

You can see dirt, dust, and fibre particles everywhere in the room. As there is no source of ventilation in the room, the air and the floor remain full of fibre particles and dust, which becomes problematic for us in the long run. **Female Participant 8**

We have a very restricted space in which we are compelled to work. It is not more than 35 inches (88.9 cm). It is difficult to keep the room clean. **FG3**

These narratives show that carpet weavers are forced to work in unclean and inadequate spaces without lighting, ventilation, and toilet facilities.

### Experiences related to designing of tools

**Figure 2.**
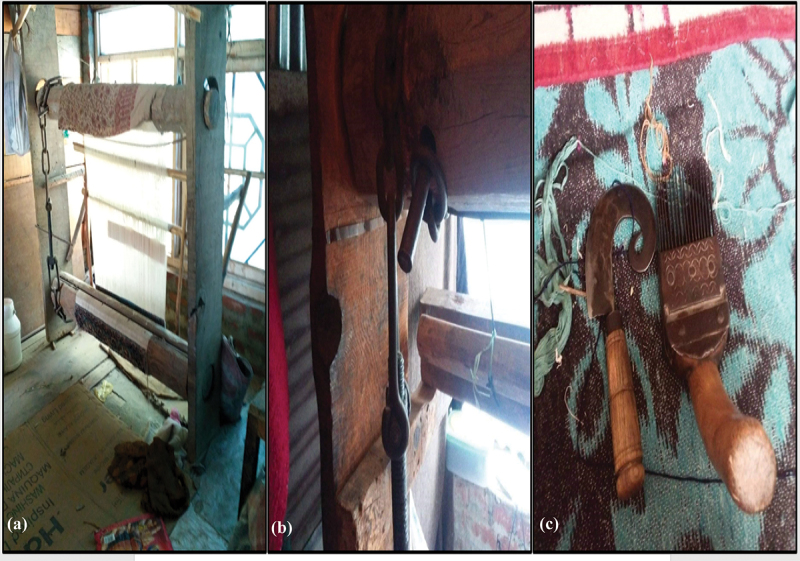
Figure 2 (a) depicts a wooden loom; Figure (b) displays the chain of the loom; Figure (c) shows khoor and panje.

Source: fieldwork

**Figure 3.**
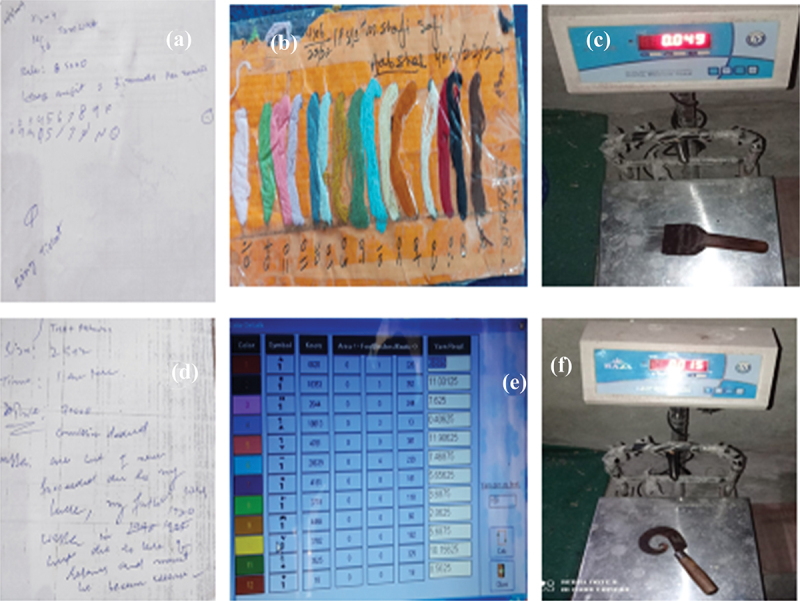
(a & d) represents field notes (b) represents handmade rang ticket and (e) denotes computer designed rang ticket, and (c & d) represents weight of the weaving tools.

Source: fieldwork

In all the workshops, most of the participants were quite dissatisfied with the tools they had to use in the weaving process like the traditional wooden looms, *khoor* (curved knife), *panje* (comb), skewer, chain, *kamaan*, etc. (see Figure 2a–c). They were dissatisfied with the shape and weight of these instruments as they were difficult to use and handle. The tools used in carpet weaving are hefty and difficult to carry. The weight of a curved knife *(khoor)* ranged between 90 gm to 150 gm, while the weight of the comb *(panje)* was 490 gm (see Figure 3c,f); also see (Khan, 2022). The quality of the wool/silk determines the weight of the tool required to weave the carpet. As revealed by many participants, the use of these tools resulted in physical exertion and injuries.

The curved knife proves disastrous at times as there are always chances of cuts and bruises. I also got a deep cut some days back while using the knife. The combs we use to compress each weaved row are very heavy, and, given its weight, using it is uncommonly laborious. **Female participant 5**

One day during work, I was exhausted, and suddenly the knife slipped from my hand and fell on my left knee. The tip of the knife caused a hole at the upper side of the knee, and blood started oozing out from the wound. The wound was so deep that it took me almost three weeks to recover. **Male Participant 2**

From these narratives, we can say that participants voiced significant dissatisfaction with the tools they were required to use in the carpet weaving process. Many participants revealed that prolonged use of these tools led to physical exhaustion and even injuries, further complicating the already challenging task of weaving.

### Experiences related to computerized taleem

**Figure 4.**
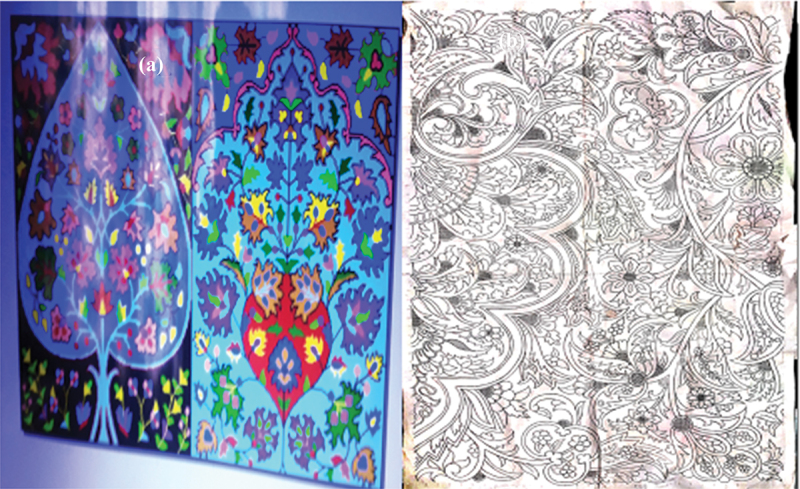
(a) represents CAD; Figure (b) displays hand-made design.

Source: fieldwork

The change from the traditional handwritten *taleem* (coded instructions for weaving carpets) to the computerized *taleem* is highly challenging for the weavers in Kashmir (see Figure 4a,b). Most participants were dissatisfied with the computerized *taleem*. The old handwritten *taleem* was easy to read and comprehend as the font of the *taleem* and the space between letters was satisfactory. The carpet weavers were able to read it from even a distance. However, the computerized *taleem* comes in a small font that forces the weavers to get closer to reading it. The change in *taleem* is not only affecting the eyesight of the weavers but also results in investing almost double the time on making the same carpet.

The taleem written by hand was overall, good and did not affect my eyes. Since the computerized taleem is intricate and printed in small font, reading it requires more effort on my part. My eyes have experienced severe issues as a result. The doctors have advised me to wear glasses during work. **Male Participant 4**

The use of computer-aided designs has ruined the work of local designers/taleem writers. No one among us now knows the art of indigenous writing. **FG3**

These quotes reflect that the transformation from handwritten *taleem* to computer-designed taleem is beneficial for the owners and customers but not for the carpet weavers.

### Nature of relationship with wastas

We also found that most participants were quite dissatisfied with their masters. The participants were dissatisfied with the wages their *wasta* gave them for making carpets; they were not given the wages they deserved. Participants also narrated that *wasta* does not provide the money on time, and they have to work very hard to get their hard-earned money from the *wasta*, sometimes leading to intense arguments. Moreover, *wasta* reduced the wages if the weaver failed to finish the carpet on time or had made any mistake in weaving the carpet. The *wasta* does not pay heed to their demands like replacing old or defective equipment and paying for holidays.

The wages my wasta gives me to complete this carpet are very low. For the last carpet, he gave me 15,000. Later, I learned that he had sold the same carpet for 50,000. This is a grave injustice to weavers like me. **Male Participant 7**

The unruly behavior of *wastas* and intermediaries causes frustration in numerous ways. For instance, *wastas* and intermediaries pick up faults resulting in wage reduction; they give post-dated cheques that often bounce. This we only get to know from bank authorities at the time of withdrawal. So far, three of us have filed a case in the District Court to recover the amount. FG1 & FG1*

The above quotes narrative the nature of the relationship between participants and their *wastas* (masters) is characterized by significant dissatisfaction. Participants consistently expressed frustration with the unfair wages they received for their labour-intensive work in carpet making, stating that they were not compensated, as they deserved. Adding to their grievances, the *wasta* showed little regard for their requests, such as replacing old or faulty equipment or providing payment for holidays, leaving the weavers feeling exploited and undervalued.

#### Health issues experienced by carpet weavers

Participants in our study discussed many health challenges they face at their workplaces. Often stated were their exposures to health hazards, both visible and invisible. During interviews and participant observation, we observed that they work in *tin-sheds* or the rooms constructed on the cowshed. Most of these workplace units have a working environment that is unsafe and unhealthy for the workers. The workers primarily work in small, dark, and crowded rooms with unsuitable furniture, improper ventilation and lighting, and no efficient safety measures in case of emergencies. In such an environment, carpet weavers face a myriad of health challenges, encompassing musculoskeletal problems, occupational injuries, respiratory issues, eye problems, and stress (see Table III).

**Table III.** Major health issues faced by participants.

Health issues	Male (n=13)		Female (n=9)		Total (N=22)	
	F	%	F	%	F	%
Musculoskeletal problem	13	100.00	9	100.00	22	100.00
Respiratory problem	10	76.92	6	66.67	16	72.73
Skin problem	9	69.23	6	66.67	15	68.18
Eye problem	7	53.85	5	55.56	12	54.55
Ear problem	6	46.15	4	44.44	10	45.45
Fatigue	9	69.23	7	77.78	16	72.73
Stress	12	92.31	5	55.56	17	77.27
Injuries	5	38.46	3	33.33	8	36.36
Others (Abdominal pain, nose problem, anemia)	4	30.77	2	22.22	6	27.27

Source: fieldwork

N (number), F (frequency), % (percentage)

### Musculoskeletal problems experienced by carpet weavers

Almost all the participants reported some type of musculoskeletal complaints in their different body parts like neck, shoulders, lower and upper back, hands, ankles, and knees. They attributed these health issues to their inappropriate sitting postures as they mostly work while sitting on the floor in a hunched position with folded legs (see Figure 1a,b).

I often feel pain in my whole body, mostly in the areas around the neck and knees. As the workplace is crammed up, we need to sit on the floor with folded legs. We also have to move our necks up and down continuously. Working continuously in such a posture caused severe problems in my knees. The problem turned so grave that doctors asked me to go for surgery on my left knee, or my leg would become paralyzed forever. **Male Participant 10**

I work almost 14 hours every day. I start at 8 in the morning and stop working at 11 at night. I only leave my workplace at the time of Lipton chai (tea), lunch, and Noon chai (Kashmiri local tea). Other than these, I do not take any breaks. Working so long has caused disc problems in my back. These days I work while wearing a belt. **Female Participant 9**

*The above narratives reflect the prevalence of musculoskeletal problems among the carpet weavers.*

### Occupational injuries experienced by the carpet weavers

**Figure 5.**
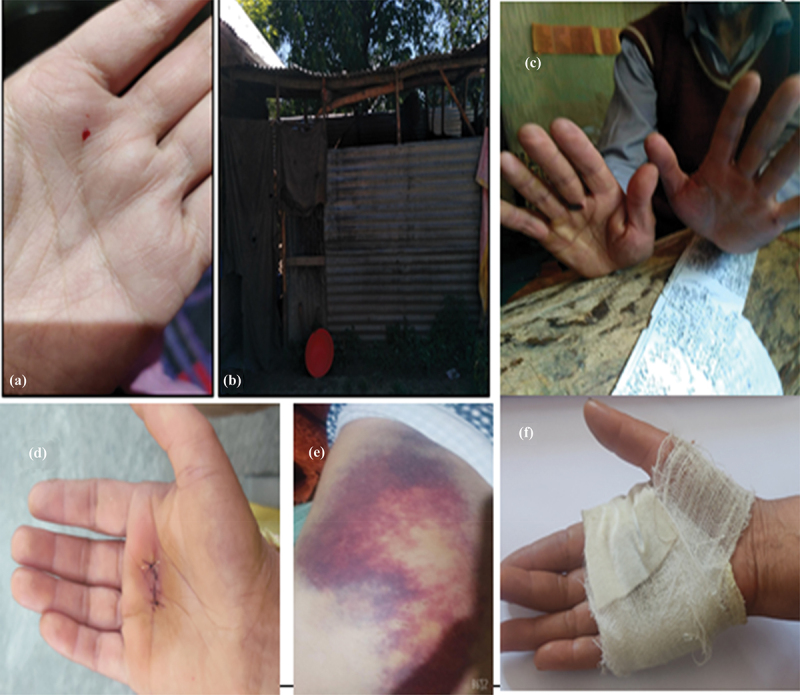
(a) displays the cuts during weaving; Figure (b) displays the tin shed (sometimes used for weaving and some for cowshed).

Source: fieldwork

The participants also revealed that apart from suffering from different musculoskeletal problems, they were also prone to injuries, hand tendons, and bruises due to imbalance while handling tools, physical as well as mental exertion, and the continuous use of *khoor* (curved knife) during their work (see Figure 5). Continuous use of the curved knife also increased the chances of wrist sprains.

Due to lengthy workdays and the intervention of master weavers, we frequently suffer from loom-related wounds, bruises, and injuries. When a skilled weaver smacked me in the face for a mistake I made while weaving a carpet one day, the knife flew from my hand and landed on my right foot. The knife’s tip cut the upper side of the foot, and blood began to ooze from the wound. I needed over a week to heal because the wound was so severe. **Male Participant 13**

We often face toe, finger and hand injuries in the looms. The most dangerous injury is the head injury by the loom. **FG3**

We have been working in this workshop from past four years and in total we have above ten years work experience. Most of us have developed tendon in either the right or left hand. I was recently hospitalized for the treatment of tendon release tenotomy.

These narratives identify the occupational injuries faced by the carpet weavers due to long working hours and traditional tools.

### Respiratory problems experienced by the carpet weavers

The majority of the participants also reported respiratory problems like coughing, sneezing, nausea and breathing problems. They attributed these problems to exposure to dust and fibre particles generated during weaving, unhygienic rooms, poor ventilation, and over-crowdedness. It was found that while weaving carpets, the air in the working place area remained filled with cotton, wool, and silk fluffs. During the participant observation, all the workers were found working without using masks, so these tiny particles were breathed in all the time causing, in the end, asthma and tuberculosis. They also highlighted that chemicals added to the threads during the dyeing process stay on them, which cause inflammation and allergy in the lungs. The problem was severe among those who worked on looms at home and had to sleep in the same room at night.

I have been doing this work for the last 30 years. I used to have an acute cough due to the dusty environment and constant intake of fibre particles. At first, I took it lightly and thought I would be fine in a few days. However, after some days, I had difficulty breathing and severe chest pain. I then decided to visit the Chest Disease Hospital in the Dalgate area of Srinagar. After the proper medical examination, I was diagnosed with asthma. **Male Participant 17**

The disastrous floods in 2014 completely damaged our house. Since we had no resources to construct a new house, we made this tin shed. It has only one room with a loom fixed on one end. We cook, eat, and even sleep in this very room only. We both my husband) and (me suffer from chronic bronchitis with a persistent cough. **Female Participant 15**

From these narratives, we can say these respiratory problems such as cough, sneezing, nausea, and breathing is common among the participants in the study.

### Eye problems faced by carpet weavers

Another major health problem revealed by our participants was related to their eyes. Weaving a carpet is an intricate craft that needs uninterrupted concentration to make delicate and exact knots. In order to reach the desired level of perfection, a proper lighting system is required. However, it was observed that the workshops lacked the desired lighting system. The participants also highlighted that the frequent power cuts (mostly during winters) and non-availability of an inverter aggravates their problem as they are forced to work using candles, *lalteen* (traditional Kashmiri Lanterns), and modern chargeable lanterns. Especially among elderly weavers, working under dim light worsened their eyesight, resulting in blurring of vision.

The workshop lacks a proper lighting facility. The room in which we weave carpets requires at least six bulbs. However, at present, only two bulbs are installed there. It is even more challenging when we are forced to work under candles and lanterns during power shutdowns. I am suffering from poor eyesight because of this inadequate lighting issue. Doctors have warned me that I can lose eyesight in both my eyes if the problem persists. **Male Participant 22**

The continuous reading from the computerized *taleem* also affected their eyes. It was revealed that the font of the computerized *taleem* was much smaller than the handwritten *taleem*. To clearly read from the *taleem* in smaller font and understand it correctly, they need to look at the *taleem* paper with more concentration, resulting in considerable eyestrain. Redness, inflammation, and irritation in the eyes were also prevalent among them due to the fibre and dust particles that remain all over the workplace.

Handwritten taleem was, to a large extent, better and was not affecting my eyes much. The computerized taleem is complex and written in small font, so I need to focus more on reading it. It has created serious problems for my eyes. I have to use glasses while working. **Female Participant 3**

One day, after finishing my work, my right eye turned red and itchy. After a few minutes, my eye was so swollen I could hardly see. I went to an ophthalmologist for consultation. After check-ups and tests, I was told that the dust and fibre particles had accumulated in my eyes. The doctors will now perform a minor surgery for both my eyes. **Female Participant 15**

### Factors reinforcing stress among carpet weavers

During data collection, we also realized that the participants suffered from stress and depression. The main reasons for stress were the clashes with *wasta* (master), working conditions, life events, work-life balance, and the time limit given by the *wasta* to finish weaving the carpet. Participants revealed that they are supposed to finish the carpet on the due date. If they fail to do so, there are wage cuts. There are wage cuts if the *wasta* finds any fault in the carpet or figures out that they have not done their work according to the given *taleem*. This further leads to stress among the weavers. Participants also revealed that they take the advance from the *wasta*. Then from time to time, they borrow from their *wasta* and are unable to repay the entire amount. So they work like bonded labourers, and even if they wish, they cannot work for another *wasta* until they clear their debts. This way, they get trapped in a vicious cycle.

My sister is getting married in a few months. I do not have enough money to pay for her wedding expenses. The wages given by my wasta are low, and he has not given me any money for the last seven months. I am in stress. Sometimes I feel gabrahath (nervousness). Although I am physically at my workplace, mentally I am somewhere else. **Male Participant 7**

I was supposed to complete this carpet in eight months. After I finished half of it, my husband fell from a walnut tree and underwent multiple surgeries. Due to his illness, I could not finish the carpet on time. Being stressed, I even made some mistakes in weaving. Although I explained everything to him (wasta), he decreased my wages by 40 per cent. It was very disturbing for me. For the last three months, I have been on anti-depressants. **Female Participant 21**

Another reason for stress for these weavers was the future of their children. Immersed in debt and without economic resources, they were not able to educate their children like others in their communities. They feared their children would also be forced to enter the same job, which would turn their future dark, just like theirs.

We do not earn much from this job. On an average, I earn Rs. 150 per day. With this, I am not able to meet the daily needs of my family. The biggest concern for me is that I am not able to send my children to school. I cannot even pay for their uniform, books, and other items. Their future is dark, and I feel guilty for that. **Male Participant 20**

The narratives under this theme reflect that financial strain works as a significant stressor. Many carpet weavers took advances from their *wastas* and frequently borrowed money, which they struggled to repay. This financial dependency often left them in a precarious situation where they worked under conditions akin to bonded labour. The inability to clear their debts prevented them from seeking work with other *wastas*, trapping them in a continuous cycle of financial and emotional stress. These factors collectively contribute to a high level of stress and depression among carpet weavers, impacting their overall well-being and quality of life. Addressing these issues is essential for improving their mental health and working conditions and for fostering a supportive and fair working environment in the carpet weaving industry.

## Discussion

This study offers new perspectives on how poor workplace conditions affect carpet weavers’ health in many ways. The following discussion examines weavers’ accounts of their working conditions in light of the study’s goals based on a qualitative theoretical framework. Given that this study employs qualitative methods of inquiry, in this discussion, we place marked emphasis on prospects for policy development aimed at enhancing working conditions and lowering occupational vulnerabilities for weavers. Weavers are at risk of various harms (harm capturing more than the presence of disease including occupational injuries due to the use of potentially heavy instruments and occupational diseases due to extended workdays and inappropriate work postures. This study will help researchers find potential risk factors by focusing on the infrastructural facilities in the weaving stations. It will enable public health policies to focus on prevention and the importance of preventive measures to promote health care among weavers. The current study shows that carpet weavers work in a poor working environment as our findings showed that majority of the participants were dissatisfied with their working environment (such as noise level, thermal condition, lighting condition, workstation design, tools, and cleanliness of the air), limited space, and design (as previously proposed by Nazari et al., 2012).This limited physical space in the weaving station influences women’s mental health and work efficiency more than men. Consistent with previous studies, the present study highlights that most participants were either extremely dissatisfied or somewhat satisfied with the seating arrangement (Choobineh et al., 2004; Mahmoudi & Bazrafshan, 2015). Motamedzade (2009) reported that in carpet weaving workshops, the vertical looms were placed in such a way that the weaver was not able to move his/her legs and was forced to work in fixed or less flexible leg positions. This inability of leg movements caused joint pain and stress over different body parts, thus making these health issues a part of the everyday lives of weavers. The results also indicated that most participants were dissatisfied with the lighting condition of the workshops, therefore providing evidence to support research findings from other scholars; for instance, Wani and Jaiswal (2012) also highlighted that the lighting condition of carpet units was substandard. The mean illumination level in all seasons was low compared to the standard level of 500 lx, resulting in eye irritation, eyestrains, and forced harmful postures due to poor lighting. Participants were also dissatisfied with the quality of air in the workshops. The participants revealed that weaving generated dust, wool, and cotton particles, which, inhaled during breathing, caused severe respiratory problems. Consistent with the findings of the previous studies (Choobineh et al., 2004; Motamedzade, 2009; Nazari et al., 2012), the results of our study revealed that participants were not satisfied with the tools they used in weaving carpets. They reported issues with the shape and weight of the hand tools, causing musculoskeletal problems. Apart from these working conditions highlighted by previous studies, our study also found that participants were dissatisfied with the computerized *taleem* and their relationship with their master. These two are the unique findings of this study. Our study highlights the importance of considering how rapidly evolving technologies, (modern designs) are used in weaving stations with a perspective to support and encourage the production and consumption of woven goods. However, in reality, it creates challenges (joblessness for *taleem* writers, the requirement of spending more time on weaving, stress, alienation) for the weavers.

The findings highlight multiple occupational health problems that prevail in the hand-woven carpet industry in Kashmir. Most of these health problems result from a poor working environment focusing less on safety and health (Awan et al., 2010; Choobineh et al., 2004; Wani et al., 2015). Our study revealed that carpet weavers suffered from more than five health problems. In line with the previous studies (Choobineh et al., 2007; Karimi et al., 2016; Mahdavi et al., 2018; Mohammadpour et al., 2018; Nazari et al., 2012; Wani & Jaiswal, 2012; Wani et al., 2015), we found that carpet weavers suffered from musculoskeletal problems and were prone to injuries due to the nature of their work. Nazari et al. (2012) highlighted that the prevalence of musculoskeletal complaints was considerably high in almost all the body parts of the hand-woven carpet weavers. We found that such musculoskeletal problems were directly related to their inappropriate sitting postures, tools used for weaving, and long working hours. Motamedzade (2009) argued that the seating arrangement, poor design of hand tools, and no space for leg movements resulted in high incidences of musculoskeletal injuries among weavers. We also found that respiratory diseases were common among carpet weavers. Due to the unclean environment and the constant exposure to contaminated air filled with fibre particles, they suffered from acute cough, asthma, tuberculosis, nausea, and other respiratory ailments. This is consistent with the studies conducted by other researchers (Bhat & Rather, 2009; Ozesmi et al., 1987; Sharma et al., 2014; Wani & Jaiswal, 2012). Choobineh et al. (2004) revealed that due to poor ventilation, the wool fibres produced and released during wool preparation, combing knots, and cleaning process exposed weavers to different respiratory diseases. In their study, Gani and Shah (1998) reported that asthma and primary tuberculosis were most prevalent among child carpet weavers due to the contaminated air in the workshops. The blurring of vision, loss of eyesight, and eye allergies were also common health problems among the participants in our study. Some previous studies also supported this finding (Wani et al., 2015; Yekta et al., 2011). Choobineh et al. (2004), in their study, stated that inadequate lighting resulted in considerable eyestrains among carpet weavers. Gani and Shah (1998) revealed that due to the dusty environment, watering, irritation, and redness of the eyes were common complaints among carpet weavers. However, the unique finding of our study, which is not highlighted by any previous study, is the impact of frequent power cuts (mainly in the winter season when transmission lines get damaged due to heavy snowfall and rains) and the impact of computerized taleem on the eyesight of the carpet weavers.

We also observed that the female carpet weavers experienced more occupational stress than the male carpet weavers did. Samani& Ghaljahi, (2018) also revealed that female carpet weavers had a high level of occupational stress owing to the prevalence of different musculoskeletal disorders. Awan et al. (2010) documented common psychosocial risks and work-related stress among carpet weavers. Working children in their study had many health complaints that could reflect social and psychological stresses. However, the results of our study are different from these studies in the sense that stress was caused by many reasons like low wages, clashes with the master, people’s envy and evil eye, deadlines to complete work, change from handwritten taleem to computerized *taleem*, and debt.

The findings from this study suggest that policymakers should consider the potential risks imposed by poor working conditions on carpet weavers. The weavers need to be recognized as workers who deserve special care. The lack of recognition may also be due to a lack of public awareness of the risks associated with poor working conditions for carpet weavers. Informal workers, particularly carpet weavers, should be recognized as vulnerable to the technological advances of weaving products on the one hand and occupational health hazards owing to poor working conditions on the other hand. While there has been a focus on marketing (export productivity) in the carpet industry literature, public health researchers and policymakers also need to monitor the range of practical measures (offering free medical camps for weavers, adequate working facilities) that may facilitate and encourage weavers to engage in carpet manufacturing without facing occupational vulnerabilities. This will be important in ensuring that weavers and other informal workers are protected from any risks from poor working conditions. Public education must also emphasize craft education to raise awareness among policymakers of the health concerns connected with unsafe working conditions. Finally, our study suggests that improvement in the design of the looms and tools and the proper seating arrangement can reduce their exposure to musculoskeletal problems. Proper and adequate lighting facilities should be made available so that the eyesight of the carpet weavers is not affected. There is a need for training programmes regarding precautionary measures and safety during work. There should be regular monitoring of the safety and health at workshops to check the working conditions. Information about policies and programmes must be disseminated to provide a safe working environment or health allowances. One such scheme includes the Health Insurance Scheme designed on the footprints of the Rashtriya Swasthya Bima Yojana (RSBY) by Ministry of Labour and Employment. The scheme aims to give the weaver community the financial means to use the nation’s best medical facilities. The plan would cover the weaver, his wife, and their two children for any new illnesses while keeping a sizable portion set aside for OPD. Finally, weavers need to change their perception about the understanding of exposure to hazards and measures to minimize them. These steps would improve the condition of weavers working in the various household or industrial units in Kashmir and help them make an effective contribution to the economy of Kashmiri society.

## Policy interventions and implications

This research offers valuable insights for policymakers aiming to enhance workplace safety for carpet weavers in Kashmir. By implementing action research, we can empower these workers to address occupational risks and improve their working conditions. It is essential to include restrooms in each workshop to cater to the basic needs of the weavers.

Moreover, public health policies should focus on improving infrastructural aspects, such as better seating arrangements, adequate lighting, and proper ventilation, to prevent health issues proactively. Recognizing that weavers face multiple health problems, policies should target the root causes related to their work environment. The study also highlighted a lack of awareness among weavers regarding occupational risks. Therefore, it is crucial to ensure that they have access to protections and support from the formal health sector and are included under industrial health policies and programmes.

## Conclusion

This study aimed to understand the everyday life experiences of carpet weavers in Kashmir, with a focus on how poor working conditions impact their health. The findings from the field revealed that a high prevalence of musculoskeletal problems, including issues in the neck, shoulders, knees, wrists, elbows, and both upper and lower back. We also identified a range of respiratory problems such as cough, cold, sneezing, asthma, and tuberculosis; eye issues including visual impairment, allergies, redness, and inflammation; as well as skin conditions like callosities and other allergies. Participants reported significant fatigue and stress, with stress linked to low wages and adverse behaviour from *wastas* (including wage reductions and criticism of work), while fatigue was credited to long working hours and inadequate infrastructure.

The study highlights the connection between these health problems and poor working conditions, such as improper sitting postures, substandard tools, and the psychological strain from environmental and social factors like people’s envy and insecurity in Kashmir. Addressing these issues is crucial not only for improving the health and well-being of the weavers but also for preserving Kashmir’s carpet industry and ensuring the sustainability of their livelihoods.

However, while this study provides valuable insights, its scope was limited to weavers working on vertical traditional wooden looms with 18 colours. Therefore, the results may not be fully applicable to weavers using horizontal looms or mixed modern and traditional looms, nor to those working with a broader range of colours. Further research is needed to explore these aspects and to develop comprehensive strategies for improving working conditions across different weaving settings. By addressing these challenges, it is possible to enhance the well-being of carpet weavers and contribute to the preservation and growth of the region’s renowned carpet industry.

## Notes

1. *Taleem* is a coded script in which one unit of code represents several knots and colour code. It is a Kashmiri innovation (Harris, 2001; Kour, 2023).

2. The term *“Ustaad”* denotes a master or highly skilled weaver with exceptional expertise in carpet weaving. An *Ustaad* often plays a pivotal role in training and mentoring younger or less experienced weavers, imparting their extensive knowledge and skills.

3. In Kashmir, the term “*Shagirds”* refers to apprentices or students learning the craft under the guidance of a master weaver, known as an Ustaad. The term *“Shagird”* is derived from Persian, meaning “disciple” or “learner.”

4. The term *“Kalbaaf”* refers to a highly skilled weaver who specializes in creating intricate and complex patterns within the carpet. The term *“Kalbaaf”* is derived from Persian, with “kal” meaning “pattern” or “design” and “baaf” meaning “weaver.” A *Kalbaaf* is an expert in weaving the detailed and often elaborate designs that are characteristic of Kashmiri carpets.
